# Construction Notes

**Published:** 1893-04-15

**Authors:** 


### CONSTRUCTION NOTES.

A house near the Devon and Exeter Hospital, and in connection with the same, is to be temporarily rented and furnished to provide additional accommodation for twelve more nurses to send to private cases.

At the annual meeting of the Aberdeen Dispensary and Lying-in Institution it was suggested that a house should be rented in the vicinity of the Dispensary for the purpose of a maternity hospital.

